# Building, Baking, and Finishing an Inlay

**Published:** 1905-09

**Authors:** L. A. O’Brian

**Affiliations:** New York


### BUILDING, BAKING, AND FINISHING AN INLAY.[1]

BY DR. L. A. O'BRIAN, NEW YORK.

Gentlemen of The New York Institute of Stomatology,—For many years I have been a member of this society, but visit it to-night for the first time. I wish to thank the society for the honor conferred upon me by the committee in asking me to read a paper on a subject in which I have had many years experience; and although I have had the pleasure of demonstrating before many of you, I feel I shall still be able to offer you something new.

My part of the programme to-night is to tell you of my methods

---

[1] Read before The New York Institute of Stomatology, May, 1905.

of building, baking, and finishing the inlay, and so I will devote myself to these subjects, avoiding digressions as much as possible in the hope that the discussions will give me opportunity to go into details which a ten-minute paper does not permit.

I commence by making a statement, which I hope in the discussion to demonstrate, by telling of my first experiments in producing a so-called low-fusing porcelain thirteen years ago, and also by offering fused samples of some porcelains for your inspection.

My statement is that so-called low-fusing bodies are glass with all its objectionable weaknesses, while the high-fusing bodies are strictly porcelain. I shall confine myself to operations with the latter, and must, in consequence, assume that the matrix is platinum.

This matrix I lay carefully in a platinum tray, in which I have an ample quantity of finely powdered asbestos moistened with alcohol.

After shaking the matrix well into the asbestos, I take a spatula and cover the edges of my matrix to prevent any jar or accident from contorting it; and this tray I now place on the front of the muffle of my Hammond furnace to dry, while I am preparing my body.

I take Close's body for the reason that it has, when fused, the closest approach to the color of the dentine of the human tooth and forms a background such as you will find in the human tooth when denuded of its enamel.

By this time the asbestos is dry, and having moistened the porcelain body with alcohol, on account of its evaporating qualities, I begin to build my porcelain inlay by first dropping into the matrix a drop of alcohol, which acts as a vehicle to float the porcelain body into every crevice of the impression.

Great care is taken to prevent the slightest overflow over the edge, and, as the building proceeds, I bring the moisture to the surface by drawing a file backward on the handle of my melting-pan, absorbing it with small strips of blotting-paper. This is now placed in front of the muffle to dry, care being taken not to hurry it, for nothing kills the success of a porcelain operation more surely than to attempt to hurry any part of it, although the temptation to do so is almost irresistible.

When dry the pan is carefully set into the slightly warmed muffle and the heat is gradually increased to the highest point of the rheostat. What this temperature is I do not know and do not care, for I never depend upon the pyrometer, but prefer to trust to my more experienced eye. After thoroughly fused, I cut off the current and allow the inlay to remain in the muffle until the glow has disappeared in order not to disturb molecular arrangement by a too sudden change of extreme temperature. On removal I have a foundation no more translucent than dentine itself and resembling that tissue very much. Now we come to the question of color, and here I use the low-fusing bodies which can, when so used, be properly called porcelain enamels.

Ash & Sons make the best as far as material is concerned, but the assortment of colors leaves much to be desired, and here Jenkins enamel helps one out.

The objection to any low-fusing body is, that although you may decide upon a sample which exactly matches the tooth which you propose to restore, if your inlay does not have the same thickness as the sample the color will be different. Put more clearly, I mean to say, that one-half dozen pieces of varying thicknesses made from the same shade and melted together under exactly the same condition, will be of six different shades, all of which will be again changed when the cement is put on.

I am desirous of being very explicit, but some things are easier to demonstrate than to explain, as experience must govern the operation.

I take a shade, or a mixture of shades, much more intense than the shade of the tooth, and cover my inlay with an even coating of it and melt. The body of my inlay is opaque, as is the dentine of a tooth, while the glassy enamel carrying the coloring material is semi-translucent, as is the enamel of a tooth. In no other way can you produce a filling which looks the same from all sides, and you can discard the precarious layer experiments and your cement does not change the appearance of the inlay after setting.

We come now to the last step, the insertion of the inlay. I never use the dam in a porcelain operation, especially if the cavity approaches the gingival margin, but by napkins and cotton rolls I keep the saliva from the tooth and dry it thoroughly with warm air. Ames's cement I use exclusively, and mix it just thick enough to be able to retain it as a globule, which does not drop from the spatula, and this is wiped into the cavity and poked about until every part of the cavity is covered and all air-bubbles have been expelled. The back of the inlay is also carefully covered by my assistant meanwhile, after which I place it in the cavity, pressing it home with a piece of smooth, thin tape, which I remove with a drawing motion which wipes away the overflow of cement. My assistant now hands me a piece of waxed floss silk, about one-quarter yard long, and this I wind in a peculiar manner around tooth and inlay, and this silk, by its elasticity, forces every particle of excessive cement out and holds the inlay securely in place until crystallization has been completed. The more thoroughly to protect the operation, I drop melted paraffine over tooth and inlay, and allow my patient to sit in the waiting-room for from fifteen to twenty minutes, when I remove the silk, and the operation is done. A filling should never be ground because, first, if carefully made by an experienced operator, it will not be necessary, and secondly, if it is ground, one changes the surface angles of refraction by so doing, and in some lights the filling or inlay will look changeable.

My mental picture of this operation has for some reason or other taken the form of a corner to be put upon an incisor tooth, let us say a central, and as the man who first produced a low-fusing porcelain (so-called) and who for thirteen years, in a busy practice, became proficient, if I may be so egotistical, let me say that I never produced, or saw produced, with a gold impression and glass body, any such operations as can be produced in the manner which I have described, and the operator will be spared the shame of failures, especially in such operations as involve incisor corners, which, with low-fusing bodies, show after a few days of use a crevice or depression, caused by chipping of the inlay, on the incisive edge which can be entered by the finger-nail.

Glass, as we all know, is dense and hard, but it is also friable, although it may stand a great strain under slow, crushing force, it will not stand a grinding force, especially on incisive corners, where it must form a perfect right angle with the enamel of the tooth at its incisive edge.

Consequently, I advocate the high-fusing, stronger bodies for such cases.

The ten minutes allowed must by this time have expired, and thanking you for your attention, I shall be pleased to answer any questions and also to explain the different samples I have with me should they prove of interest to any of the gentlemen present. Jenkins's fuses at 1552°, Parker's at 2588°, Close's at 2300°.

